# ﻿New records of water mites (Acari, Hydrachnidia) from Portugal revealed by DNA barcoding, with the description of *Atractidesmarizae* sp. nov.

**DOI:** 10.3897/zookeys.1151.100766

**Published:** 2023-03-01

**Authors:** Vladimir Pešić, Milica Jovanović, Amália Espiridião Oliveira, Ana Pedro, Marvin Freira, Maria Manuela Morais

**Affiliations:** 1 Department of Biology, University of Montenegro, Cetinjski put b.b., 81000 Podgorica, Montenegro; 2 Mediterranean Institute for Agriculture, Environment and Development (MED), CHANGE – Global Change and Sustainability Institute, Institute for Advanced Studies and Research, Universidade de Évora, Pólo da Mitra, Ap. 94, 7006-554, Évora, Portugal; 3 Water Laboratory, University of Évora, P.I.T.E. Rua da Barba Rala No. 1, 7005-345 Évora, Portugal; 4 Institute of Earth Sciences–ICT, University of Évora, Rua Romão Ramalho 59, 7000-671, Évora, Portugal; 5 Department of Biology, School of Sciences and Technologies, Pólo da Mitra Apartado 94 7002-554, Évora, Portugal

**Keywords:** New records, new species, systematics, taxonomy

## Abstract

This study presents the first results of DNA barcoding of water mites from Portugal. DNA barcodes were recovered from 19 water mite specimens morphologically assigned to eight species, seven of them newly reported from Portugal. Two species, *Torrenticolahispanica* (Lundblad, 1941) and *A.cultellatus* (K. Viets, 1930) were discovered more than 80 years after they were first described, and *Atractidesmarizae***sp. nov**. is described as new for science.

## ﻿Introduction

The water mites of Portugal are still insufficiently known. Water mites of mainland Portugal and its archipelagos (Madeira and Azores) were studied by Barrois (1887, 1896), Koenike (1895), Thor (1898), Viets (1918), Lundblad (1941, 1942, 1954, 1956), and Cantallo et al. (2021, 2022). The most recent check list of water mites of Portugal and its archipelagos was published by Cantallo et al. (2022), who reported 93 hydrachnid species from 34 genera and 16 families. All of these species were exclusively identified on the basis of morphological characters, and until now there have been no studies analyzing the genetic diversity of this important but often neglected limnofaunistic group.

In recent years, the use of the mitochondrial cytochrome c oxidase subunit I (COI) gene, has proven to be a highly effective tool for delimiting and identifying water mites, in particular for recognizing species complexes with potential cryptic diversity (Martin et al. 2010; Pešić et al. 2012, 2017, 2019, 2020, 2022; Fisher et al. 2017; Pešić and Smit 2020). The use of this system, known as DNA barcoding, in recent taxonomic studies has been accelerated by the formation of worldwide databases for the storage and public identification of sequences, such as GenBank and the BOLD system (DNA Barcode of Life Data System).

In some regions, COI data on water mites has been intensively accumulated in recent years and has led to the compilation of national and regional DNA barcode libraries (e.g., Blattner et al. 2019; Pešić et al. 2021a, b; Pešić and Smit 2022). This has enabled a better assessment of the molecular diversity of water mites in specific habitats, as well as the identification of problematic species groups, resulting in the description of a number of cryptic or pseudocryptic species that would probably remain undescribed using solely classical taxonomic methods.

The main aim of this study is to enrich the existing reference library with new sequences of specimens collected in Portugal and present the taxonomic results of this collecting effort.

## ﻿Materials and methods

Water mites were collected by hand netting, sorted live from other organisms and debris in the field, and immediately preserved in 96% ethanol for the purpose of the molecular analyses (see below). Water-mite specimens used for the molecular study are listed in Table 1. After DNA extraction, the specimen vouchers were stored in 96% EtOH and morphologically examined. Some of these vouchers were dissected and slide mounted in Faure’s medium, while the rest was transferred to Koenike’s fluid and stored in the collection of the first author. DNA sequences prepared in the course of this study were deposited in BOLD and GenBank. The DNA extracts were archived in −80 °C freezers at the Centre for Biodiversity Genomics (**CBG**; https://biodiversitygenomics.net).

**Table 1.** Details on barcoded specimens, including data and coordinates of sampling sites, the barcode index number (^N^ indicates a new BIN that contains only current sequences) and associated data obtained from BOLD. DNN = distance to nearest neighbor; NN BIN = nearest neighbor BIN; NN taxonomy = species assigned to nearest neighbor BIN. BOLD data presented here was last accessed on 10 January 2023.

Species	Locality	Coordinates	Voucher Code	BOLD/GenBank Acc Nos	BIN BOLD	DNN (%)	NN BIN BOLD:	NN taxonomy
**Lebertiidae**								
*Lebertiapusilla*	Santarém, Caniceira	39.4110°N, 8.2615°E	CCDB_39397_B06	HYDAS018-22/OQ211647	–	–	–	–
			CCDB_39397_C03	HYDAS027-22/OQ211648				
**Torrenticolidae**								
*Torrenticolahispanica*	Santarém, Caniceira	39.4110°N, 8.2615°E	CCDB_39397_B10	HYDAS022-22/OQ211664	^N^ BOLD:AES2742	14.02	BOLD:AEW2607	*Torrenticola* sp.
*Monatractidesstadleri*	Beja, Corgo da Ponte Quebrada	37.6961°N, 8.7122°E	CCDB_39397_B05	HYDAS017-22/OQ211649	BOLD:AEU1504	8.98	BOLD:AED3802	*Monatractidesstadleri*
**Oxidae**								
*Oxusangustipositus*	Porto, Silveirinhos	41.1727°N, 8.5007°E	CCDB_39397_A06	HYDAS006-22/OQ211652	^N^ BOLD:AET9442	5.59	BOLD:AED9576	*Oxusangustipositus*
			CCDB_39397_A08	HYDAS008-22/OQ211651				
			CCDB_39397_A07	HYDAS007-22/OQ211650				
**Hygrobatidae**								
*Atractidesmarizae* sp. nov.	Santarém, Caniceira	39.4110°N, 8.2615°E	CCDB_39397_B12	HYDAS024-22/OQ211637	^N^ BOLD:AER7878	12.98	BOLD:AEN9154	*Atractidesgiustinii*
			CCDB_39397_C04	HYDAS028-22/OQ211643				
			CCDB_39397_C05	HYDAS029-22/OQ211642				
			CCDB_39397_C02	HYDAS026-22/OQ211640				
*Atractidesallgaier*	Beja, Corgo da Ponte Quebrada	37.6886°N, 8.7043°E	CCDB_39397_B02	HYDAS014-22/OQ211639	^N^ BOLD:AEU1287	14.58	BOLD:ACS0163	*Atractidesdistans*
			CCDB_39397_A09	HYDAS009-22/OQ211641				
*Atractidescultellatus*	Santarém, Caniceira	39.4110°N, 8.2615°E	CCDB_39397_B11	HYDAS023-22/OQ211638	^N^ BOLD:AEU1503	16.01	BOLD:ADG8744	*Atractidesrivalis*
**Pionidae**								
*Pionanodata*	Herdade do Pinheiro	38.4953°N, 8.7097°E	CCDB_39397_C06	HYDAS030-22/OQ211655	^N^ BOLD:AET0101	10.43	BOLD:ACR9882	*Pionanodata*
			CCDB_39397_C07	HYDAS031-22/OQ211656				
			CCDB_39397_C08	HYDAS032-22/OQ211657				
			CCDB_39397_C09	HYDAS033-22/OQ211653				
			CCDB_39397_C10	HYDAS034-22/OQ211654				

Morphological nomenclature follows Gerecke et al. (2016). The genital acetabula in both sexes and the genital plate in the female were measured on both sides; therefore, their dimensions are given as a range of values, rather than a single value. The holotype and paratypes of the new species are deposited in the Naturalis Biodiversity Center in Leiden (**RMNH**).

All measurements are given in μm. The photographs of selected structures were made using the camera of a Samsung Galaxy smartphone. The following abbreviations are used: **Ac-1** = first acetabulum; **Cx-I** = first coxae; **Dgl-4** = dorsoglandularia 4; **dL** = dorsal length; **H** = height; **I-L-4-6** = fourth-sixth segments of first leg; **L** = length; **lL** = lateral length; **mL** = medial length; **P-1-P-5** = palp segment 1-5; **S-1** = proximal large ventral seta at I-L-5; **RMNH** = Naturalis Biodiversity Center, Leiden; **S-2** = distal large ventral seta at I-L-5; **Vgl-1** = ventroglandularia 1; **W** = width.

### ﻿Molecular and DNA barcode analyses

The molecular analysis was conducted at the Canadian Centre for DNA Barcoding (Guelph, Ontario, Canada; CCDB; http://ccdb.ca/). The specimens were sequenced for the barcode region of COI using standard invertebrate DNA extraction (Ivanova et al. 2007), amplification (Ivanova and Grainger 2007a), and sequencing (Ivanova and Grainger 2007b) protocols.

DNA barcode sequences were aligned using MUSCLE alignment (Edgar 2004). Primer nucleotide sequences were removed, and chromatograms were checked for the presence of double peaks, stop codons, and frameshifts, which could indicate the amplification of nuclear mitochondrial pseudogenes. None of the DNA sequences showed evidence of pseudogenes.

Data related to each BIN, including the minimum *p*-distance to the nearest neighboring BIN, was estimated through BOLD. Intra- and interspecific genetic distances were calculated based on the *p*-distance model using MEGA X (Kumar et al. 2018). MEGA X software was used to calculate neighbour-joining (NJ) trees based on K2P distances (standard for barcoding studies) and pairwise deletion of missing data. The support for tree branches was calculated by the nonparametric bootstrap method (Felsenstein 1985) with 1000 replicates and shown next to the branches. Codon positions included were 1^st^+2^nd^+3^rd^+Noncoding.

## ﻿Results and discussion

This study represents first DNA barcodes of water mites from Portugal with a COI barcode dataset obtained from 19 specimens and morphologically assigned to six genera (Table 1). The two species, *Torrenticolahispanica* (Lundblad, 1941) and *Atractidescultellatus* (K. Viets, 1930), which are both endemic to the Iberian Peninsula, were uploaded into the BOLD database; these contribute to the formation of a DNA barcode reference library for the reliable identification of water mite species in future studies. Moreover, one species is described as new for science, and seven species are reported as new for water-mite fauna of Portugal.

### ﻿Description of new species

#### ﻿Family Hygrobatidae Koch, 1842

##### ﻿ Atractides (Atractides) marizae

Taxon classificationAnimaliaTrombidiformesHygrobatidae

Pešić sp. nov.

2F33991C-CCC1-52E8-A76B-C03973020118

https://zoobank.org/97384632-7c6e-4387-9a59-d6d907670250

Figs 1 , 2A–D , 3

###### Type material.

***Holotype*** ♂ (sequenced, CCDB_39397_C02, Table 1), dissected and slide mounted (RMNH), Portugal, Santarém, Caniceira stream, 39.4110°N, 8.2615°W, 25.v.2022 leg. Jovanović. ***Paratypes***: 3♂, 2♀, same site and data as the holotype, 2♂, 1♀ sequenced (Table 1), 1♂ (CCDB_39397_C0) damaged (one palp and I-legs missing), 1♀ (CCDB_39397_B12) dissected and slide mounted (RMNH).

###### Diagnosis.

Characters of the *nodipalpis*-species group (integument finely striated, muscle insertions unsclerotized; males with anteriorly and posteriorly indented genital field, P-2 with distoventral projection and ventral margin of P-4 projecting); excretory pore smooth, acetabula relatively small, arranged in an obtuse triangle.

###### Description.

**Figure 1.**
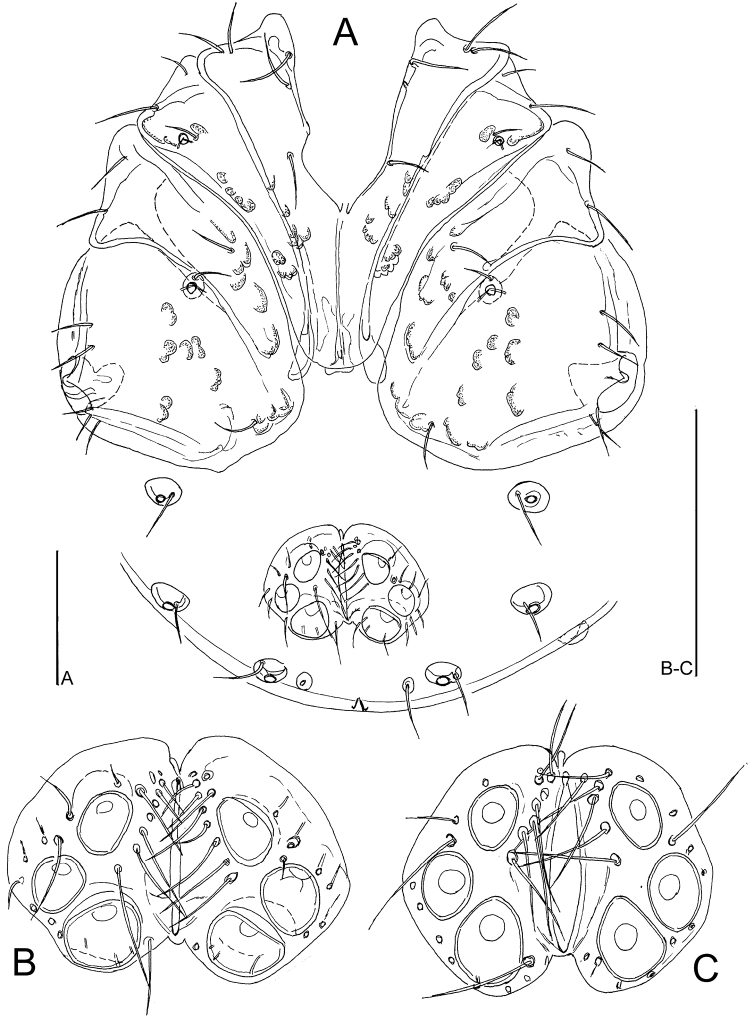
*Atractidesmarizae* nov. sp., ♂ **A, B** holotype, CCDB_39397_C02 **C** paratype, CCDB_39397_C04 **A** idiosoma in ventral view **B, C** genital field. Scale bars: 100 µm.

**Figure 2.**
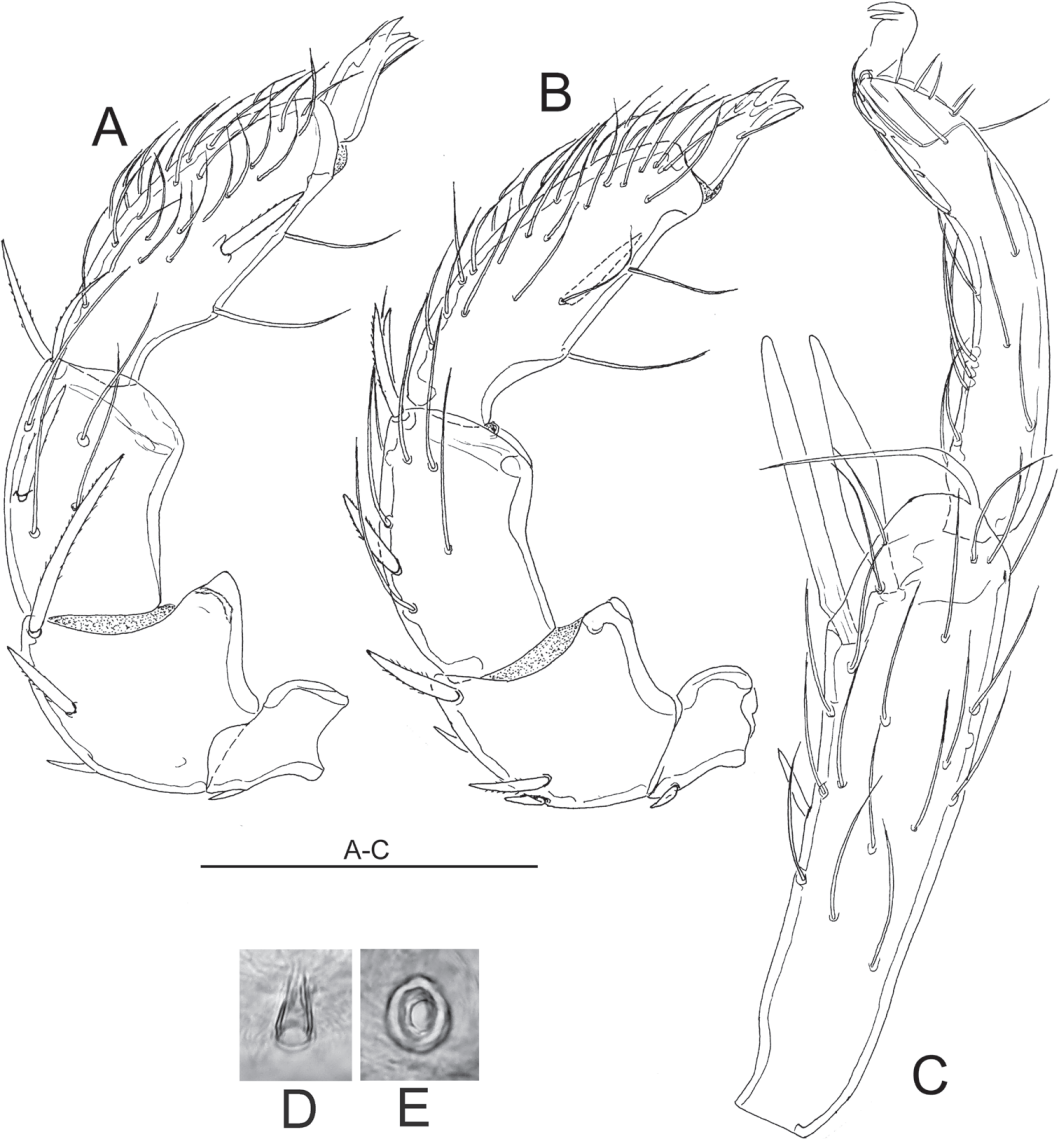
**A–D***Atractidesmarizae* nov. sp., ♂ holotype, CCDB_39397_C02 **A** palp in medial view **B** palp in lateral view **C** I-L-5 and -6 **D** excretory pore **E***A.ruffoi*, ♀ CCDB_39397_C02, Corsica; excretory pore. Scale bar: 100 µm.

**Figure 3.**
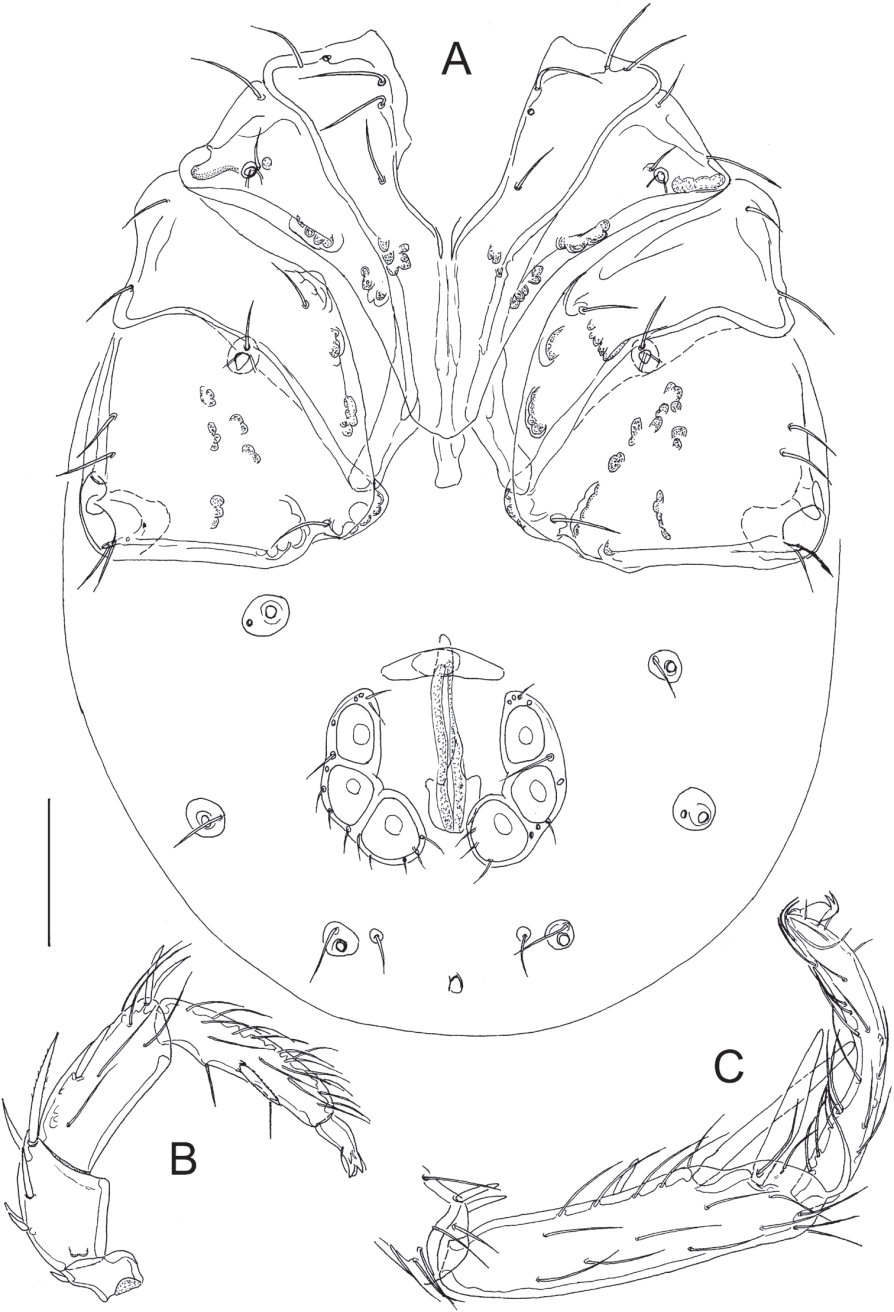
*Atractidesmarizae* nov. sp., ♀ paratype, CCDB_39397_B12 **A** idiosoma in ventral view **B** palp in medial view **C** I-L-5 and -6. Scale bar: 100 µm.

***General features***–Integument striated, muscle insertions unsclerotized; mediocaudal margin Cx-I strongly convex, apodemes of Cx-II in an acute angle with the median line. Excretory pore smooth; Vgl-1 not fused to Vgl-2. Palp with strong sexual dimorphism in shape of P-2 and P-4, in both sexes medial peg-like seta inserting halfway between ventral setae, seta insertions dividing ventral margin into three equal sectors. I-L-5 proximally subrectangular, distally protruding near insertion S-1, with seta S-1 slender and bluntly pointed, S-2 shorter and pointed, proximally enlarged; I-L-6 slender, curved, basally slightly thickened from the centre to the claw furrow with parallel dorsal and ventral margins (Figs 2C, 3C). **Male**–Anterior margin of genital plate with a notch and bead structure, a fine median tip projecting in a deep indentation; caudal margin with a deep indentation extending to about 1/2 L of Ac-3, Ac rounded to subtriangular, arranged in an obtuse triangle (Fig. 1B, C); ventral margin P-2 with a strongly developed distal extension, P-3 strongly concave, P-4 proximally concave, inflated near proximoventral seta. **Female**–Caudal apodemes of Cx-I +II strongly protruding, Cx-IV with well-developed apodemes at medial margins (Fig. 3A), P-2 nearly straight with a right-angled ventrodistal edge, P-3 dorsal margin slightly concave, P-4 more slender than in the male (Fig. 3B).

###### Measurements.

**Male** (holotype, CCDB_39397_C02; in parentheses some measurements of paratype, CCDB_39397_C04)–Idiosoma L 559 (538), W 458 (425); maximum diameter Dgl-4, 28. Coxal shield L 344 (303); Cx-III W 388 (334); Cx-I+II mL 117 (122), Cx-I+II lL 244 (206). Genital field L/W 91(94)/129(117), L Ac-1-3: 25–28 (25–28), 23–27 (26–30), 30–31 (32–34). Ejaculatory complex L 94.

Palp–Total L 338; dL/H, dL/H ratio: P-1, 31/30, 1.05; P-2, 73/58, 1.26; P-3, 83/45, 1.83; P-4, 111/41, 2.73; P-5, 40/14, 2.8; L ratio P-2/P-4, 0.66. Gnathosoma vL 125, chelicera total L 222.

Legs–I-L-5 dL 195, vL 142, dL/vL ratio 1.37, maximum H 49, dL/maximum H 3.99, S-1 L 98, L/W ratio 10.5, S-2 L 78, L/W ratio 4.99, distance S-1-2, 16, dL ratio S-1/2, 1.26; I-L-6 dL 141, central H 22, dL/central H ratio 6.46; L I-L-5/6 ratio 1.38.

**Female** (CCDB_39397_B12)–Idiosoma L 686, W 531. Coxal shield L 369; Cx-III W 466; Cx-I+II mL 122, Cx-I+II lL 263. Genital field L/W 150/167, genital plates L 122–124, pregenital sclerite 84, gonopore L 119, L Ac-1-3: 41, 39–41, 42.

Palp–Total L 454; dL/H, dL/H ratio: P-1, 38/38, 1.02; P-2, 97/64, 1.51; P-3, 127/52, 2.43; P-4, 147/36, 4.09; P-5, 45/19, 2.41; L ratio P-2/P-4, 0.66. Gnathosoma vL 158, chelicera total L 280.

Legs–I-L-5 dL 277, vL 194, dL/vL ratio 1.43, maximum H 66, dL/maximum H 4.22, S-1 L 145, L/W ratio 12.8, S-2 L 114, L/W ratio 6.1, distance S-1-2, 36, dL ratio S-1/2, 1.27; I-L-6 dL 202, central H 22, dL/central H ratio 9.22; L I-L-5/6 ratio 1.37.

###### Etymology.

The new species is named in honor of Marisa dos Reis Nunes, known professionally as Mariza, a famous Portuguese fado singer in the appreciation of the enjoyment her music brings to the authors.

###### Species delimitation using DNA-barcodes.

**Figure 4.**
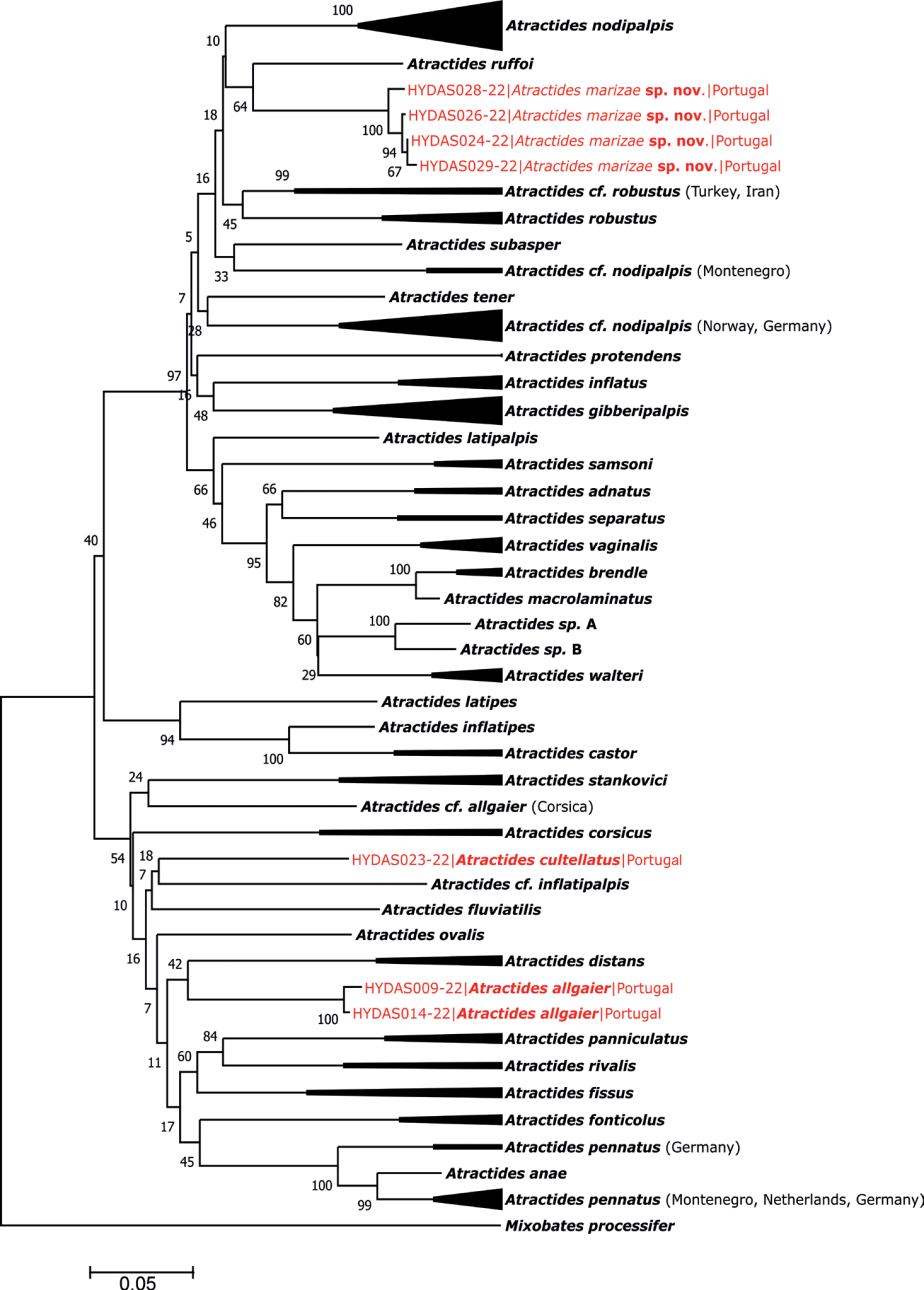
Neighbour-joining tree of the genus *Atractides* obtained from 175 nucleotide COI sequences.

The final alignment for species delimitation using COI sequence data comprised 674 nucleotide positions (nps) of the 175 *Atractides* specimens, morphologically assigned to 40 species listed in Suppl. material 1 and one outgroup, *Mixobatesprocessifer* from Norway to root the tree. The NJ tree is presented in Fig. 4. The COI tree sequences retrieved from specimens of *A.marizae* sp. nov. from Portugal appeared as a sister clade of *A.ruffoi* Gerecke & Di Sabatino, 2013, a rhitrobiontic species endemic to Corsica (Gerecke and Di Sabatino 2013). The *p*-distance between the COI sequences of specimens of *A.marizae* sp. nov. from Portugal and one specimen of *A.ruffoi* from Corsica was estimated at 13.34 ± 1.3%, indicating genetic separation between these two clades. The mean intraspecific divergence within the clade of new species from Portugal was relatively low (1.09 ± 0.27).

###### Remarks.

Pešić and Smit (2022), by mistake, assigned the voucher specimen (CCDB 38559A09) of *Atractidesruffoi* from Corsica to *A.giustinii* Gerecke & Di Sabatino, 2013, a species endemic to Corsica and Sardinia. Therefore, the sequence NOVMB009-21/ON002561 deposited in BOLD/GenBank belongs to *A.ruffoi*.

###### Discussion.

In regard to the striated integument, a characteristic “notch and bead” structure of male genital field, and the shape of the palp in the male (P-2 with distoventral projection, ventral margin of P-4 projecting), the new species resembles *A.nodipalpis* Thor, 1899, *A.robustus* (Sokolow, 1940), and *A.ruffoi*. Both sexes of *A.nodipalpis* and *A.robustus* differ by having larger acetabula in a triangular arrangement. *Atractidesruffoi* differs by the development of a sclerite at the excretory pore (Gerecke and Di Sabatino 2013).

A characteristic “notch and bead” structure of the male genital plate is found also in *A.clavipalpis* (Lundblad, 1956), which in males, differ from the new Portuguese species in having the ventral margin of P-2 distally slightly protruding and not forming a projection, and a distally club-shaped P-4 (Gerecke 2003).

###### Habitat.

**Figure 5.**
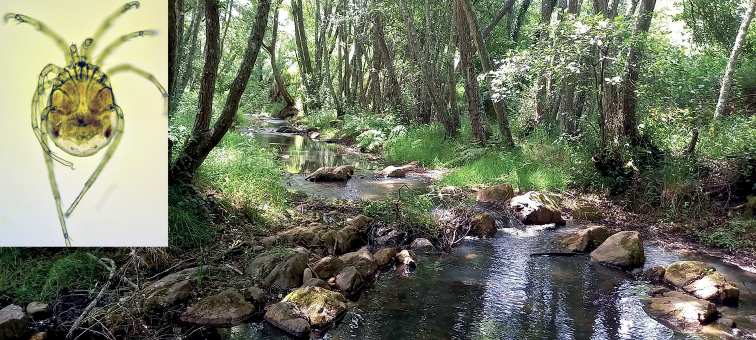
Photograph of locus typicus (Caniceira stream, Santarém, Portugal) of *Atractidesmarizae* sp. nov. (inset). Photographs by M. Jovanović.

A rhithrobiont. Collected in a low-order stream, with shaded pool reaches having accumulations of leaf litter (Fig. 5).

###### Distribution.

Portugal; only known from the type locality.

### ﻿Species new for water mite fauna of Portugal

#### ﻿Family Lebertiidae Thor, 1900

##### ﻿ Lebertia pusilla

Taxon classificationAnimaliaTrombidiformesHygrobatidae

Koenike, 1911

3870C319-5A1A-5997-AA43-0033807720C7

###### Material examined.

Portugal, Santarém, Caniceira stream, 39.4110°N, 8.2615°W, 25.v.2022, leg. Jovanović, 2♂, 4♀, 2♀ sequenced (Table 1).

###### Remarks.

The Portuguese specimens molecularly analyzed in this study match the description of *L.pusilla*, a species widely distributed in the Palaearctic (Di Sabatino et al. 2010). They share the presence of only one short swimming seta on II-L-5 and two or three swimming setae on anterior IV-L-5. It is likely that the lineage from Portugal represents a cryptic species, with a *p*-distance of 9.39–9.79% to the nearest sequence (NLACA493-15) of *L.pusilla* from the Netherlands.

###### Distribution.

Europe.

#### ﻿Family Oxidae K. Viets, 1926

##### ﻿ Oxus (Oxus) aff.angustipositus

Taxon classificationAnimaliaTrombidiformesHygrobatidae

K. Viets, 1908

C2B0EEDA-2D66-594E-99D2-11DADC8ABE1A

###### Material examined.

Portugal, Porto, Silveirinhos stream, 41.1727°N, 8.5007°W, 25.v.2022, leg. Jovanović, 1♂, 2♀ (sequenced; Table 1).

###### Remarks.

The Portuguese specimen molecularly analyzed in this study matches the description of *O.angustipositus*. These individuals form a unique BIN (BOLD:AET9442), with the nearest neighboring BIN being OLD:AED9576, which consists of a specimen from Lake Ohrid, North Macedonia. The *p*-distance between the specimens from Portugal and GenBank *O.angustipositus* (Montenegro; OL870273, OL870215, OL870142, OL870101) is 8.7–9.3%; this demonstrates the need for taxonomic revision of the *O.angustipositus* complex for identifying possibly undescribed cryptic species.

###### Distribution.

Western Palaearctic.

#### ﻿Family Torrenticolidae Piersig, 1902

##### ﻿ Torrenticola (Torrenticola) hispanica

Taxon classificationAnimaliaTrombidiformesHygrobatidae

(Lundblad, 1941)

0D379FCA-83FA-5682-AD8C-DC06467AD6C8

Fig. 6

###### Material examined.

Portugal, Santarém, Caniceira stream, 39.4110°N, 8.2615°W, 25.v.2022, leg. Jovanović, 1♂, (sequenced; Table 1), dissected and slide mounted (RMNH).

###### Remarks.

The Portuguese specimen molecularly analyzed in this study perfectly matches the description of *T.hispanica*, a species originally described on basis of specimens collected from a stream near Algeciras in Spain (Lundblad 1956).

###### Description.

**Figure 6.**
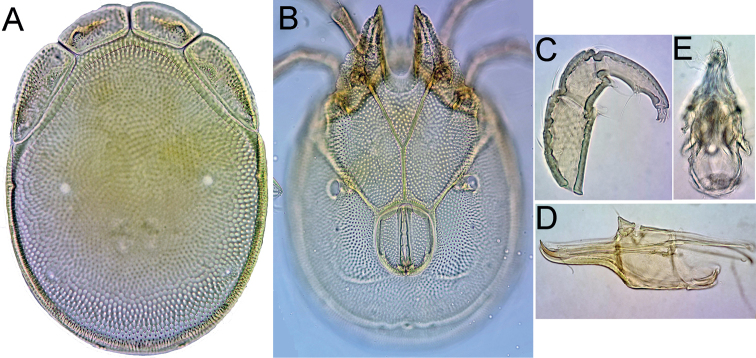
Selected parts of *Torrenticolahispanica*, ♂, CCDB_39397_B10 **A** dorsal shield **B** ventral shield **C** palp, lateral view (P-1 lacking) **D** gnathosoma and chelicera in lateral view **E** ejaculatory complex. Photographs by V. Pešić.

**Male**–Dorsal shield without a colour pattern, as shown in Fig. 6A; area of primary sclerotization of the dorsal plate with two dorsoglandularia; gnathosomal bay U-shaped, proximally rounded; Cxgl-4 subapical; suture line of Cx-IV evident, medially starting from posterior margin of genital field in a right angle to the main idiosoma axis; genital field subrectangular; ejaculatory complex conventional in shape (Fig. 6E); excretory pore located on the line of primary sclerotization; gnathosoma ventral margin curved, rostrum strongly elongated (Fig. 6D); P-2 longer than P-4; P-2 ventral margin straight, P-2 and P-3 ventrodistal protrusions blunt, laterally flattened, P-4 with a well-developed ventral tubercle bearing one longer and three shorter setae (Fig. 6C).

###### Measurements.

(CCDB_39397_B10)–Idiosoma L 784, W 572; dorsal shield L 644, W 483, L/W ratio 1.33; dorsal plate L 598; shoulder plate L 203–206, W 75–81, L/W ratio 2.54–2.71; frontal plate L 142–147, W 70, L/W ratio 2.0–2.1; shoulder/frontal plate L 1.38–1.45. Gnathosomal bay L 172, Cx-I total L 338, Cx-ImL 164, Cx-II+III mL 128; ratio Cx-I L/Cx-II+III mL 2.64; Cx-ImL/Cx-II+III mL 1.28. Genital field L/W 159/134, ratio 1.19; distance genital field-excretory pore 113, genital field-caudal idiosoma margin 156. Palp: total L 342, dL/H, dL/H ratio: P-1, 39/31, 1.25; P-2, 114/58, 1.97; P-3, 64/51, 1.26; P-4, 106/30, 3.55; P-5, 19/13, 1.5; L ratio P-2/P-4 1.08; gnathosoma vL 337, chelicera L 400.

###### Distribution.

Spain and Portugal.

##### ﻿ Monatractides (Monatractides) stadleri

Taxon classificationAnimaliaTrombidiformesHygrobatidae

(Walter, 1924)

A4F8E54F-A611-5F59-8F4E-FA98F8CF26FC

###### Material examined.

Portugal, Beja, Corgo da Ponte Quebrada, stream, 37.6961°N, 8.7122°W, 23.v.2022, leg. Jovanović, 1♂ (sequenced; Table 1), gnathosoma, palps and I-legs dissected and slide mounted (dorsal and ventral shield stored in Koenike fluid).

###### Remarks.

The Portuguese specimen molecularly analyzed in this study matches the description of *M.stadleri*, a species widely distributed in the Mediterranean region and often very frequent in lowland, running waters (Di Sabatino et al. 2010). The sequenced specimen clusters within BOLD:AEU1504, which includes two specimens of *M.stadleri* from Belgium and one specimen from Spain (identified as *Torrenticola* sp., deposited in Taxus Medio Ambiente, Spain). The *p*-distance between the latter BIN and its nearest neighbour, BOLD:AED3802, which includes specimens from Montenegro and Greece, is estimated at 8.98%. This suggests the need for taxonomic revision of the *M.stadleri* complex to identify possible undescribed cryptic species (see Pešić and Smit 2022 for a discussion).

###### Distribution.

Central, Western, and Southern Europe.

#### ﻿Family Hygrobatidae Koch, 1842

##### ﻿ Atractides (Atractides) cultellatus

Taxon classificationAnimaliaTrombidiformesHygrobatidae

(K. Viets, 1930)

788E3849-BA77-5791-BBBB-1D5796E98D63

Fig. 7

###### Material examined.

Portugal, Santarém, Caniceira stream, 39.4110°N, 8.2615°W, 25.v.2022, leg. Jovanović, 1♀ (sequenced; Table 1), dissected and slide mounted (RMNH).

###### Remarks.

The single female specimen from Portugal generally matches the description of *A.cultellatus*, which was originally described from a single female collected from Rio Manzanares, Spain (K. Viets, 1930). *Atractidesvalencianus* (K. Viets, 1930), a species originally described from Spain and later reported by Gerecke (2014) from Sardinia, resembles *A.cultellatus* in the presence of a lineated integument, a slenderer I-L-6, the more spaced sword setae of I-L-5, and Vgl-1 not fused to Vgl-2, but it differs in having P-2 completely devoid of thickening or rounding in females (Gerecke 2003).

###### Measurements.

**Figure 7.**
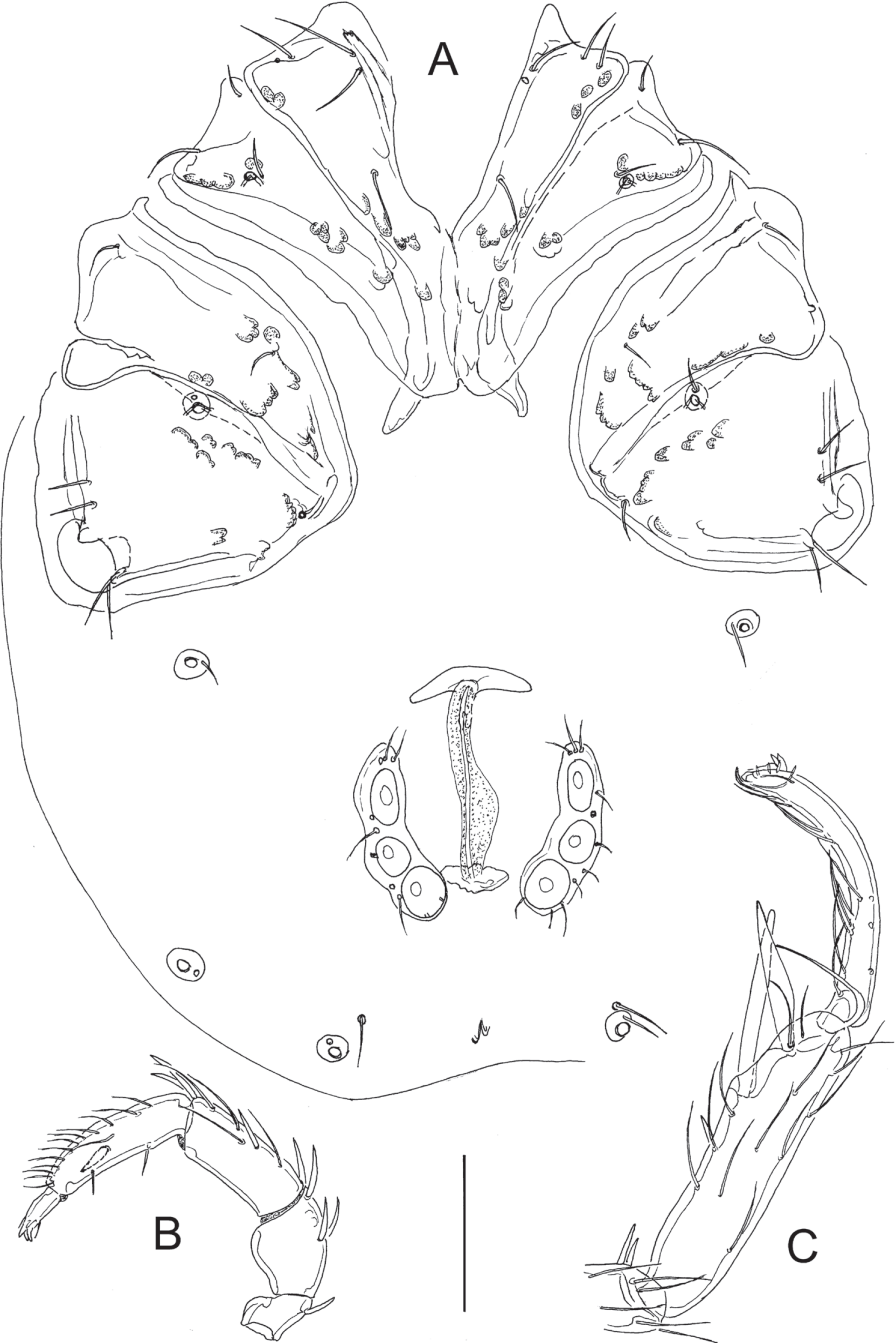
*Atractidescultellatus*, ♀, CCDB_39397_B11 **A** idiosoma in ventral view **B** palp in lateral view **C** I-L-5 and -6. Scale bar: 100 µm.

**Female** (CCDB_39397_B11)–Idiosoma L 691, W 520. Coxal shield (Fig. 7A) L 378; Cx-III W 489; Cx-I+II mL 94, Cx-I+II lL 216. Genital field L/W 163/159, genital plates L 115–118, pregenital sclerite 78, gonopore L 131, L Ac-1-3: 33–36, 28, 33. Egg maximum diemeter (*n* = 1) 147. Palp (Fig. 7B): total L 354; dL/H, dL/H ratio: P-1, 36/33, 1.1; P-2, 77/51, 1.49; P-3, 95/39, 2.43; P-4, 108/31, 3.45; P-5, 38/13, 3.0; L ratio P-2/P-4, 0.71. Gnathosoma vL 119, chelicera total L 195. Legs: I-L-5 dL 229, vL 139, dL/vL ratio 1.65, maximum H 59, dL/maximum H 3.96, S-1 L 122, L/W ratio 11.1, S-2 L 102, L/W ratio 6.5, distance S-1-2, 38, dL ratio S-1/2, 1.2; I-L-6 dL 181, central H 19, dL/central H ratio 9.63; L I-L-5/6 ratio 1.27.

###### Distribution.

Spain and Portugal.

##### ﻿ Atractides (Atractides) allgaier

Taxon classificationAnimaliaTrombidiformesHygrobatidae

Gerecke, 2003

505D4B08-ECAA-5C3D-9826-54DA0F29D7A2

###### Material examined.

Portugal, Beja, Corgo da Ponte Quebrada stream, 37.6886°N, 8.7043°W, 23.v.2022, leg. Jovanović, 2♀ (sequenced; see Table 1); Corgo da Ponte Quebrada stream, 37.6961°N, 8.7122°W, 23.v.2022, leg. Jovanović 1♀.

###### Remarks.

Populations of this species have often been confused with those of *Atractidesdistans* (K. Viets, 1914); see Gerecke (2003) for a discussion. Clear morphological differences, for example the presence of a lineated integument in *A.allgaier*, instead of striated one in *A.distans*, are confirmed with a large (>14%) *p*-distance between these species.

###### Distribution.

Central, Western, and Southern Europe.

#### ﻿Family Pionidae Thor, 1900

##### ﻿ Piona aff. nodata

Taxon classificationAnimaliaTrombidiformesHygrobatidae

(Müller, 1776)

811BEBD8-15D9-5CB8-AE79-1B558DA093A0

###### Material examined.

Portugal, Reserva Natural do Estuário do Sado, Herdade do Pinheiro, 38.4953°N, 8.7097°W, 10.v.2022, leg. Oliveira, 2♂, 2♀ (sequenced; Table 1).

###### Remarks.

The Portuguese specimens molecularly analyzed in this study match description of *P.nodata*. Genetic data indicate that all examined specimens form a cluster (BOLD:AET0101) and belong to the same species. This BIN is solely composed of the Portuguese specimens; the closest neighboring BIN is that of *P.nodata* (BOLD:ACR9882">) from the Netherlands. The high *p*-distance (10.45%) between these two BINs indicates that the Portuguese lineage may represent a cryptic species.

###### Distribution.

Holarctic.

## Supplementary Material

XML Treatment for Atractides (Atractides) marizae

XML Treatment for Lebertia pusilla

XML Treatment for Oxus (Oxus) aff.angustipositus

XML Treatment for Torrenticola (Torrenticola) hispanica

XML Treatment for Monatractides (Monatractides) stadleri

XML Treatment for Atractides (Atractides) cultellatus

XML Treatment for Atractides (Atractides) allgaier

XML Treatment for Piona aff. nodata

## Supplementary materials

Supplementary material 1 List of *Atractides* specimens used for building the neighbour-joining (NJ) tree (Fig. 4) Data type word document https://binary.pensoft.net/file/818153This dataset is made available under the Open Database License (http://opendatacommons.org/licenses/odbl/1.0/). The Open Database License (ODbL) is a license agreement intended to allow users to freely share, modify, and use this Dataset while maintaining this same freedom for others, provided that the original source and author(s) are credited.Vladimir Pešić, Milica Jovanović, Amália Espiridião Oliveira, Ana Pedro, Marvin Freira, Maria Manuela Morais
