# Supplementary material for: ﻿New records of water mites (Acari, Hydrachnidia) from Portugal revealed by DNA barcoding, with the description of Atractidesmarizae sp. nov
Source: Zookeys. 2023 Mar 1;1151:205–22. doi: 10.3897/zookeys.1151.100766 (PMC10193435; doi:10.3897/zookeys.1151.100766)
Supplement: Supplementary material 1 — List of Atractides specimens used for building the neighbour-joining (NJ) tree (Fig. 4) [file zookeys-1151-205_article-100766__-s001.doc]

**Supplementary material.** List of *Atractides* specimens used for building the Neighbour-Joining (NJ) tree (Fig. 4).

| **Taxon** | **BOLD Acc Nos** | **Voucher Code** | **Country** | **BIN BOLD** |
| --- | --- | --- | --- | --- |
| *Atractides nodipalpis* | NLACA993-17 | RMNH.5070751 | Netherlands | ACR0209 |
|  | MMHYD081-19 | HYDCA215 | N Norway |  |
|  | DCCDB034-21 | CCDB38233 C10 | Serbia |  |
|  | NLACA452-15 | RMNH.ACA.878 | Netherlands |  |
|  | HYDCA065-15 | HYDCA65 | Greenland |  |
|  | HYDCA069-15 | HYDCA69 | Norway |  |
|  | NLACA943-17 | RMNH.5070701 | Netherlands |  |
|  | NLACA1121-17 | RMNH.ACA.1536 | Netherlands |  |
|  | MMHYD038-19 | HYDCA172\| | Norway |  |
|  | MMHYD080-19 | HYDCA214 | Norway |  |
|  | DNCBD090-20 | CCDB-3867-H06 | Serbia |  |
|  | DCCDB033-21 | CCDB38233 C09 | Serbia |  |
|  | DNCBD067-20 | CCDB-3867-F07 | Montenegro |  |
|  | NLACA991-17 | RMNH.5070749 | Netherlands |  |
|  | NLACA1118-17 | RMNH.ACA.1533 | Netherlands |  |
|  | HYDOC024-22 | CCDB 44300 B12 | Serbia |  |
|  | MMHYD162-19 | HYDCA296 | Norway |  |
|  | NLACA1120-17 | RMNH.ACA.1535 | Netherlands |  |
|  | NLACA1053-17 | RMNH.5070811 | Netherlands |  |
|  | NLACA1084-17 | RMNH.5091736 | Netherlands |  |
|  | MMHYD444-21 | HYDCA578 | Germany |  |
|  | MMHYD443-21 | HYDCA577 | Germany |  |
|  | NLACA408-15 | RMNH.ACA.829 | Netherlands |  |
|  | NLACA992-17 | RMNH.5070750 | Netherlands |  |
|  | NLACA1119-17 | RMNH.ACA.1534 | Netherlands |  |
|  | MMHYD066-19 | HYDCA200 | Norway |  |
|  | DNAEC072-20 | 41. M19 29A 1 G5 | Montenegro |  |
| *Atractides ruffoi* | NOVMB009-21 | CCDB 38559 A09 | France | AEN9154 |
| *Atractides marizae* **sp. nov.** | HYDAS028-22 | CCDB 39397 C04 | Portugal | AER7878 |
|  | HYDAS026-22 | CCDB 39397 C02 | Portugal |  |
|  | HYDAS024-22 | CCDB 39397 B12 | Portugal |  |
|  | HYDAS029-22 | CCDB 39397 C05 | Portugal |  |
| *Atractides cf. robustus* | IRANM012-20 | 19. IR17 2017 H10 | Iran | AED3548 |
|  | DCDDJ059-21 | CCDB 38361 E11 | Türkiye | AEK3669 |
|  | DCCDB065-21 | CCDB38233 F05 | Iran | AEI1810 |
| *Atractides robustus* | DNCBD048-20 | CCDB-3867-D12 | Montenegro | ADZ9348 |
|  | DNCBD047-20 | CCDB-3867-D11 | Montenegro |  |
|  | DCDDJ086-21 | CCDB 38361 H02 | Montenegro |  |
|  | DCCDB013-21 | CCDB38233 B01 | Montenegro |  |
|  | DNCBD046-20 | CCDB-3867-D10 | Montenegro |  |
|  | CCDB-3867-D10 | CCDB 41824 A10 | Bosnia |  |
| *Atractides subasper* | HYDBH064-22 | CCDB 41824 F04 | Italy | AES6460 |
| *Atractides cf. nodipalpis* (Montenegro) | DNAEC071-20 | 40. M19 29A 1 G4 | Montenegro | AED3547 |
|  | DNCBD065-20 | CCDB-3867-F05 | Montenegro |  |
| *Atractides tener* | HYDCA104-18 | HYDCA104 | Norway | ACG4776 |
| *Atractides cf. nodipalpis* (Germany, Norway) | MMHYD085-19 | HYDCA219 | Norway | ADZ1306 |
|  | MMHYD129-19 | HYDCA263 | Norway |  |
|  | MMHYD086-19 | HYDCA220 | Norway | ADY7881 |
|  | MMHYD217-20 | HYDCA353 | Norway |  |
|  | MMHYD219-20 | HYDCA355 | Norway | ADP2485 |
|  | MMHYD197-20 | HYDCA331 | Norway |  |
|  | LBCWS237-19 | CrenoBarcode H534 | Germany |  |
|  | LBCWS238-19 | CrenoBarcode H535 | Germany |  |
|  | MMHYD084-19 | HYDCA218 | Norway |  |
|  | MMHYD115-19 | HYDCA249 | Norway |  |
|  | MMHYD137-19 | HYDCA271 | Norway |  |
|  | MMHYD140-19 | HYDCA274 | Norway |  |
|  | MMHYD153-19 | HYDCA287 | Norway |  |
|  | MMHYD138-19 | HYDCA272 | Norway |  |
|  | MMHYD139-19 | HYDCA273 | Norway |  |
|  | MMHYD220-20 | HYDCA356 | Norway |  |
|  | MMHYD338-21 | HYDCA472 | Norway |  |
| *Atractides protendens* | LBCWS137-19 | CrenoBarcode H336 | Germany | ADT4472 |
|  | LBCWS138-19 | CrenoBarcode H337 | Germany |  |
| *Atractides inflatus* | DCDDJ061-21 | CCDB 38361 F01 | Türkiye | ACB4677 |
|  | SEPTB060-21 | CCDB 38362 E12 | Greece |  |
|  | DCDDJ060-21 | CCDB 38361 E12 | Türkiye |  |
|  | DCDDJ058-21 | CCDB 38361 E10 | Türkiye |  |
|  | NOVMB013-21 | CCDB 38559 B01 | France |  |
|  | SEPTB021-21 | CCDB 38362 B09 | Greece | AEO3635 |
|  | DNAEC052-20 | 14. M19 12 4 E5 | Montenegro |  |
| *Atractides gibberipalpis* | HYDBH003-22 | CCDB 41824 A03 | Montenegro | AEI3946 |
|  | HYDBH004-22 | CCDB 41824 A04 | Montenegro |  |
|  | DCCDB014-21 | CCDB38233 B02 | Montenegro |  |
|  | DCDDJ031-21 | CCDB 38361 C07 | Montenegro | AEK7766 |
|  | HYDBH001-22 | CCDB 41824 A01 | Montenegro | ADU2475 |
|  | LBCWS219-19 | CrenoBarcode H508 | Germany |  |
|  | NOVMB070-21 | CCDB 38559 F10 | France | AEO4226 |
|  | NOVMB032-21 | CCDB 38559 C08 | France |  |
|  | NOVMB082-21 | CCDB 38559 G10 | France |  |
|  | NOVMB044-21 | CCDB 38559 D08 | France |  |
|  | NOVMB010-21 | CCDB 38559 A10 | France |  |
|  | NOVMB091-21 | CCDB 38559 H07 | France |  |
|  | NOVMB071-21 | CCDB 38559 F11 | France |  |
|  | NOVMB019-21 | CCDB 38559 B07 | France |  |
|  | NOVMB081-21 | CCDB 38559 G09 | France |  |
| *Atractides latipalpis* | DCDDJ073-21 | CCDB 38361 G01 | Germany | AEK4539 |
| *Atractides samsoni* | MMHYD334-21 | HYDCA468 | Norway | AEA3265 |
|  | MMHYD335-21 | HYDCA469 | Norway |  |
|  | MMHYD337-21 | HYDCA471 | Norway |  |
|  | MMHYD336-21 | HYDCA470 | Norway |  |
| *Atractides adnatus* | LBCWS126-19 | CrenoBarcode H292 | Germany | ADU1763 |
|  | LBCWS127-19 | CrenoBarcode H293 | Germany |  |
|  | LBCWS125-19 | CrenoBarcode H291 | Germany |  |
| *Atractides separatus* | LBCWS093-19 | CrenoBarcode H243 | Germany | ADV7653 |
|  | LBCWS129-19 | CrenoBarcode H311 | Austria |  |
| *Atractides vaginalis* | LBCWS029-19 | CrenoBarcode H099 | Switzerland | ADU6015 |
|  | LBCWS033-19 | CrenoBarcode H108 | Switzerland |  |
|  | LBCWS025-19 | CrenoBarcode H078 | Switzerland |  |
|  | LBCWS021-19 | CrenoBarcode H073 | Switzerland |  |
|  | LBCWS063-19 | CrenoBarcode H186 | Italy |  |
|  | LBCWS022-19 | CrenoBarcode H074 | Switzerland |  |
|  | LBCWS030-19 | CrenoBarcode H100 | Switzerland |  |
|  | LBCWS094-19 | CrenoBarcode H244 | Switzerland |  |
| *Atractides brendle* | LBCWS243-19 | CrenoBarcode H547 | Germany | ADT1697 |
|  | LBCWS244-19 | CrenoBarcode H548 | Germany |  |
|  | LBCWS236-19 | CrenoBarcode H533 | Germany |  |
|  | LBCWS235-19 | CrenoBarcode H532 | Germany |  |
| *Atractides macrolaminatus* | LBCWS178-19 | CrenoBarcode H450 | Austria | ADU8387 |
| *Atractides* sp. A *sensu* Blattner et al. 2019 | LBCWS233-19 | CrenoBarcode H528 | Germany | ADT8145 |
| *Atractides* sp. B *sensu* Blattner et al. 2019 | LBCWS234-19 | CrenoBarcode H531 | Germany | ADU6120 |
| *Atractides walteri* | LBCWS174-19 | CrenoBarcode H446 | Austria | ADV7370 |
|  | LBCWS128-19 | CrenoBarcode H304 | Austria |  |
|  | LBCWS177-19 | CrenoBarcode H449 | Austria |  |
|  | LBCWS173-19 | CrenoBarcode H445 | Austria |  |
|  | LBCWS183-19 | CrenoBarcode H459 | Austria |  |
|  | LBCWS175-19 | CrenoBarcode H447 | Austria |  |
|  | LBCWS176-19 | CrenoBarcode H448 | Austria |  |
| *Atractides latipes* | DNAEC056-20 | 18. M19 08B 7 E9 | Montenegro | AED4000 |
| *Atractides inflatipes* | DNCBD075-20 | CCDB-3867-G03 | Montenegro | AEF1144 |
| *Atractides castor* | HYDBH063-22 | CCDB 41824 F03 | Italy | AET6487 |
|  | HYDBH071-22 | CCDB 41824 F11 | Italy |  |
|  | HYDBH072-22 | CCDB 41824 F12 | Italy |  |
| *Atractides stankovici* | DNAEC020-20 | 13. CG2020 4 B10 | Montenegro | AED3550 |
|  | DNAEC021-20 | 14. CG2020 4 B11 | Montenegro |  |
|  | DCCDB032-21 | CCDB38233 C08 | Montenegro |  |
|  | DNCBD095-20 | CCDB-3867-H11 | Montenegro |  |
|  | DCCDB031-21 | CCDB38233 C07 | Montenegro |  |
| *Atractides cf. allgaier* (Corsica) | NOVMB093-21 | CCDB 38559 H09 | France | AEO6190 |
| *Atractides corsicus* | NOVMB015-21 | CCDB 38559 B03 | France | AEO5312 |
|  | NOVMB087-21 | CCDB 38559 H03 | France |  |
|  | HYDBH039-22 | CCDB 41824 D03 | Italy | AET7893 |
| *Atractides cultellatus* | HYDAS023-22 | CCDB 39397 B11 | Portugal | AEU1503 |
| *Atractides cf. inflatipalpis* | DNAEC064-20 | 29. M19 24 4 F7 | Montenegro | AED3549 |
| *Atractides fluviatilis* | DNCBD082-20 | CCDB-3867-G10 | Montenegro | AEF1143 |
| *Atractides distans* | NLACA438-15 | RMNH.ACA.864 | Netherlands | ACS0163 |
|  | NLACA439-15 | RMNH.ACA.865 | Netherlands |  |
|  | NLACA414-15 | RMNH.ACA.838 | Netherlands |  |
|  | NLACA413-15 | RMNH.ACA.837 | Netherlands |  |
|  | NLACA412-15 | RMNH.ACA.836 | Netherlands |  |
| *Atractides allgaier* | HYDAS009-22 | CCDB 39397 A09 | Portugal | AEU1287 |
|  | HYDAS014-22 | CCDB 39397 B02 | Portugal |  |
| *Atractides panniculatus* | LBCWS141-19 | CrenoBarcode H364 | Germany | ADU8027 |
|  | LBCWS145-19 | CrenoBarcode H368 | Germany |  |
|  | LBCWS144-19 | CrenoBarcode H367 | Germany |  |
|  | LBCWS143-19 | CrenoBarcode H366 | Germany |  |
|  | LBCWS142-19 | CrenoBarcode H365 | Germany |  |
| *Atractides rivalis* | HYDME026-22 | CCDB 41823 C02 | Germany | ADG8744 |
|  | HYDME017-22 | CCDB 41823 B05 | Germany |  |
|  | HYDME018-22 | CCDB 41823 B06 | Germany |  |
| *Atractides fissus* | LBCWS198-19 | CrenoBarcode H483 | Austria | ADU0063 |
|  | LBCWS199-19 | CrenoBarcode H484 | Austria |  |
|  | DCCDB015-21 | CCDB38233 B03 | Montenegro | AEI1811 |
|  | DCCDB046-21 | CCDB38233 D10 | Montenegro |  |
|  | HYDBH005-22 | CCDB 41824 A05 | Montenegro |  |
| *Atractides fonticolus* | LBCWS202-19 | CrenoBarcode H488 | Germany | ADS3489 |
|  | LBCWS203-19 | CrenoBarcode H489 | Germany |  |
|  | LBCWS201-19 | CrenoBarcode H487 | Germany |  |
|  | DCCDB020-21 | CCDB38233 B08 | Montenegro | AEI8720 |
|  | DCCDB021-21 | CCDB38233 B09 | Montenegro |  |
| *Atractides cf. pennatus* (Germany) | LBCWS220-19 | CrenoBarcode H509 | Germany | ADV9389 |
|  | HYDME006-22 | CCDB 41823 A06 | Germany | AES4768 |
| *Atractides pennatus* (Montenegro, Netherlands, Germany) | DNAEC015-20 | 3. CG2020 2 B4 | Montenegro | ADF7007 |
|  | SEPTA024-21 | CCDB 38363 B12 | Montenegro |  |
|  | SEPTA025-21 | CCDB 38363 C01 | Montenegro |  |
|  | NLACA1085-17 | RMNH.5091737 | Netherlands |  |
|  | LBCWS221-19 | CrenoBarcode H510 | Germany |  |
|  | DNAEC027-20 | 23. CG2020 9 C5 | Montenegro |  |
|  | DNAEC028-20 | 25. CG2020 9 C6 | Montenegro |  |
|  | DNAEC042-20 | 4. M19 22 1 D8 | Montenegro |  |
|  | DNAEC066-20 | 31. M19 23 1 F9 | Montenegro |  |
|  | DNAEC067-20 | 32. M19 23 1 F10 | Montenegro |  |
|  | DNCBD009-20 | CCDB-38679-A09 | Montenegro |  |
| *Atractides anae* | DNAEC014-20 | 1. CG2020 8 B3 | Montenegro | AED1201 |
| **Outgroup:**  *Mixobates processifer* | MMHYD088-19 | HYDCA222 | Norway |  |
